# A perspective on cross‐kingdom RNA interference in mutualistic symbioses

**DOI:** 10.1111/nph.19122

**Published:** 2023-07-14

**Authors:** Serena A. Qiao, Zongyu Gao, Ronelle Roth

**Affiliations:** ^1^ Department of Biology University of Oxford Oxford OX1 3RB UK

**Keywords:** arbuscular mycorrhizal symbiosis, cross‐kingdom RNAi, endosymbiosis, plant–microbe interactions, plant–pathogen interactions, RNA interference

## Abstract

RNA interference (RNAi) is arguably one of the more versatile mechanisms in cell biology, facilitating the fine regulation of gene expression and protection against mobile genomic elements, whilst also constituting a key aspect of induced plant immunity. More recently, the use of this mechanism to regulate gene expression in heterospecific partners – cross‐kingdom RNAi (ckRNAi) – has been shown to form a critical part of bidirectional interactions between hosts and endosymbionts, regulating the interplay between microbial infection mechanisms and host immunity. Here, we review the current understanding of ckRNAi as it relates to interactions between plants and their pathogenic and mutualistic endosymbionts, with particular emphasis on evidence in support of ckRNAi in the arbuscular mycorrhizal symbiosis.

**Table:** 

	Contents	
	Summary	68
I.	Introduction	68
II.	Cross‐kingdom RNA transfer in pathosystems	69
III.	The roles of sRNAs in mutualistic symbiosis	74
	Acknowledgements	77
	References	77

## I. Introduction

Their migration onto land over 450 million years ago introduced the earliest plants to a new environment abundant in potential parasitic or mutualistic microbes, which have defined substantial aspects of land plant evolution (reviewed in Delaux & Schornack, 2021). Early plants, lacking true root systems, likely relied on symbioses similar to the modern arbuscular mycorrhizal (AM) symbiosis, which augments phosphate, nitrate and water uptake from the soil, as well as aspects of pathogen defence (reviewed in Bennett & Groten, 2022). In exchange, the obligate biotrophic AM fungi rely entirely on carbon received from the host plant, primarily in the form of fatty acids (Luginbuehl *et al*., 2017; reviewed in Rich *et al*., 2017; Roth & Paszkowski, 2017). During symbiosis, the fungus penetrates the plant root and forms elaborately branched arbuscules inside the cortical cells; accompanying this process, the plant plasma membrane envelops the hyphal structure within the peri‐arbuscular membrane (PAM). This creates the peri‐arbuscular space (PAS), a shared apoplastic compartment facilitating the exchange of nutrients and signalling molecules between the PAM and the fungal arbuscular membrane (Gutjahr & Parniske, 2013; Luginbuehl & Oldroyd, 2017).

Such signalling molecules may include small RNAs (sRNAs), which may act in RNA interference (RNAi) both endogenously and in the heterospecific partner. Plant sRNAs mediating RNAi broadly fall into two classes: small interfering RNAs (siRNAs) 21–24 nucleotides (nt) in length and produced from double‐stranded precursors; and 20–22 nt micro RNAs (miRNAs) generated from double‐stranded stem‐loop precursors, which are largely transcribed from *MIR* genes. These precursors are processed by specific ribonuclease III‐like enzymes – Dicer‐like (DCL) proteins – to produce shorter double‐stranded sRNAs, from which one strand is loaded into an ARGONAUTE (AGO) protein to target complementary sequences to the sRNA. Depending on the sRNA length and AGO type, the complementary sequence can undergo a variety of processes including post‐transcriptional gene silencing through AGO‐mediated targeting of endogenous and foreign RNAs for cleavage or translational repression; recruitment of DNA methyltransferases to target DNA sequences for methylation and transcriptional gene silencing; and the recruitment of RNA‐dependent RNA polymerases to facilitate the biogenesis of secondary siRNAs (reviewed in Zhan & Meyers, 2023). Some of these sRNAs are proposed to act in cross‐kingdom RNAi (ckRNAi) between hosts and endosymbionts – a mechanism by which one organism regulates the gene expression of a heterospecific partner through the transfer of sRNAs and the co‐option of the partner's RNAi machinery. More recently, fragments of tRNAs have been demonstrated to play a similar role to other sRNAs in ckRNAi (Ren *et al*., 2019; Sánchez‐Correa *et al*., 2022), with extracellular vesicles (EVs) proposed as a mechanism of trafficking a range of sRNA species between partners (Box 1).

Box 1 **The extracellular vesicle debate**

Extracellular vesicles, the product of non‐canonical secretion mechanisms such as multi‐vesicular bodies (MVBs) or exocyst‐positive organelles (EXPOs), have long been described as a crucial infectious mechanism in microbial interactions with animal hosts, transporting critical toxins and infectious proteins (Rizzo *et al*., 2021). More recently, similar roles have been suggested for EVs in plant–microbe interactions, shown to mediate infection in both pathogenic (Cai *et al*., 2018) and mutualistic contexts, such as in the root nodulating symbiosis with *Sinorhizobium fredii* (Li *et al*., 2022). Critically, the latter saw the downregulation of defence gene expression and the upregulated expression of symbiosis‐specific transcription factors, although the mechanism by which this occurred was not elucidated. This correlates with the evidence of sRNA transport and ckRNAi in other root nodulating symbioses (Ren *et al*., 2019) to suggest an EV‐dependent mechanism of sRNA transport in this symbiosis. Currently, the mechanisms by which these EVs and their cargo are taken up by the receiving organism are somewhat unclear, although recent evidence has suggested a role for clathrin‐mediated endocytosis (CME) in the uptake of fungal EVs by the plant host in the *Arabidopsis‐B.cinerea* pathosystem (He *et al*., 2023b).

However, whether sRNAs are largely packaged inside EVs, or if the greatest proportion are found external to EVs, remains unresolved. An encapsulation mechanism has been proposed for the ckRNAi observed in pathosystems such as that of *Arabidopsis*–*B. cinerea*, where RNase protection assays were used to show that the majority of sRNAs were protected in the absence of detergent, suggesting that these were encapsulated inside EVs (Cai *et al*., 2018); EVs have also been shown to be enriched in 10–17 nt ‘tiny’ RNAs (Baldrich *et al*., 2019). Recent experiments with more stringent protease, detergent and RNase protection assays, however, suggest a predominant extra‐vesicular association between sRNAs and RNA‐binding proteins (RBPs) in the extracellular space (Zand Karimi *et al*., 2022). The significance of EVs in ckRNAi may not be entirely absent, however, as the results from Zand Karimi *et al*. represented an uninfected state rather than a pathosystem. Furthermore, additional studies suggest that these RBPs, such as AGO1 and RNA helicases, are strongly associated with the membranes of EVs when released in response to infection (He *et al*., 2021). It is possible that RBPs form part of an EV protein corona, which has been observed in mammalian EVs but remains to be described in plants (reviewed in Buzas, 2022). Similarities to this protein‐bound transport mechanism have also been documented in parasites, such as nematode worms, that use ckRNAi as a pathogenesis mechanism (Buck *et al*., 2014; Chow *et al*., 2019); this suggests that RBP‐mediated transport is a bona fide means of sRNA export in ckRNAi. Whether the same can be said for EVs remains to be seen; such confirmation or refutation would have significant implications in the study of ckRNAi in AM symbiosis, as EVs observed in the PAS have been proposed as potential sRNA carriers (Ivanov *et al*., 2019; Roth *et al*., 2019). This role of EVs is supported by TEM evidence of MVBs fusing with the PAM, as MVB‐derived EVs have been implicated in the role of sRNA delivery from plants to pathogens (Cai *et al*., 2018). Further studies, with an emphasis on more rigorous digestion assays and microscopy, will be required to identify candidate sRNAs and their localisation relative to these EVs. For a recent review, see Holland & Roth (2023).

Here, we review emerging evidence for ckRNAi as a mechanism of controlling mutualistic plant–microbe interactions, drawing from studies of both pathogenic and mutualistic symbiosis to examine the case for ckRNAi acting in the AM symbiosis.

## II. Cross‐kingdom RNA transfer in pathosystems

### Pathogen‐to‐plant communication

**Fig. 1:**
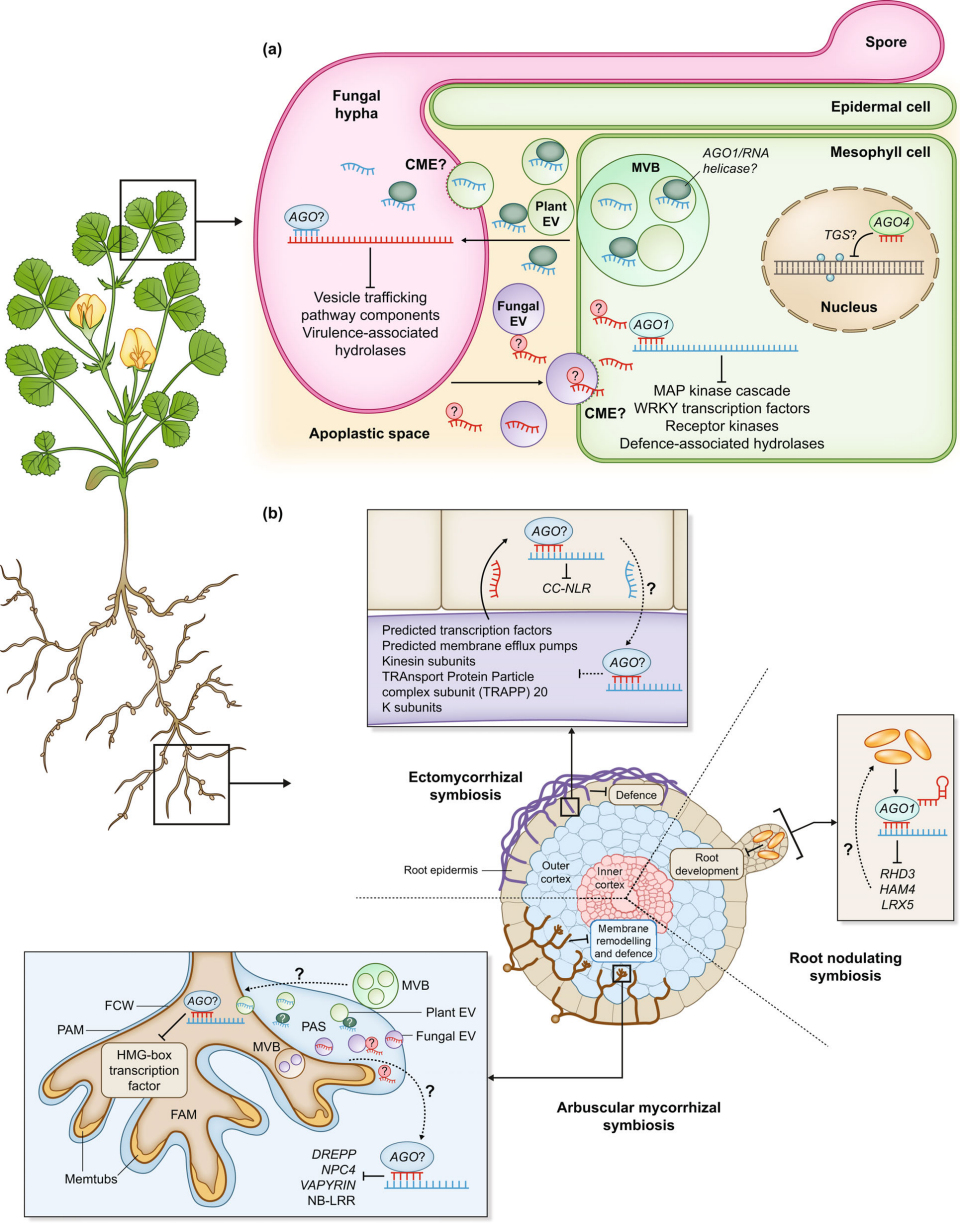
Proposed models for the functioning of ckRNAi in pathogenic and mutualistic symbiosis. (a) A representation of the ckRNAi processes occurring between the plant host and a pathogen, such as the *Arabidopsis–B.cinerea* pathosystem. Fungal components – AGO proteins, extracellular vesicles (EVs) and RNAs – are illustrated in pink and purple; plant components – AGO1 proteins, EVs, RNAs and multivesicular bodies (MVBs) – are illustrated in green and blue. Several possibilities for the transport of sRNAs are represented here (see Box 1), including the transport of sRNAs associated with RNA‐binding proteins and with some level of association with EVs (He *et al*., 2021); the fungal mechanism is largely unknown and is here represented as a mirror of proposed plant mechanisms. Question marks represent areas of uncertainty in pathogenic ckRNAi. *CME*, clathrin mediated endocytosis (b) An overview of the ckRNAi interactions that have thus far been described in mutualistic symbioses. ckRNAi has been described in root nodulating symbioses with two different bacterial strains (Ren *et al*., 2019); whilst ckRNAi in ectomycorrhizal symbiosis has currently been documented in one interaction (Wong‐Bajracharya *et al*., 2022; see Table 1 for full gene names). Note the inhibition of membrane remodelling and defence in the AM symbiosis remains to be experimentally verified (Silvestri *et al*., 2019). Memtubs – membranous tubules – are illustrated in the AM symbiosis. These have been observed between the fungal cell wall (FCW) and fungal arbuscular membrane (FAM) during symbiosis and pathogenic infections of plant tissue, and have been proposed as a source of EVs in AM symbiosis (Roth *et al*., 2019); this, however, remains unverified, and is represented by the question mark. Dotted lines and question marks represent uncertainty surrounding whether reciprocal ckRNAi from plant to mutualist occurs. *AGO*, Argonaute, *FAM*, fungal arbuscular membrane; MPA, mitogen‐activated protein; PAM, peri‐arbuscular membrane; *TGS*, transcriptional gene silencing; tRFs, red stem loop tRFs shown in root nodule.

Prediction of complementary sRNA‐mRNA sequences and cleavage assays suggest that many pathogenic heterokont and fungal species use ckRNAi to target transcripts of critical immune response genes, including mitogen‐activated protein kinase (MAPK) cascade components such as *MPK1* (Weiberg *et al*., 2013), and leucine‐rich repeat receptor kinases (LRR‐RKs) and WRKY transcription factors, such as *FEI2* and *WRKY7*, respectively (Wang *et al*., 2017; Table 1; Fig. 1a). In some instances, multiple host transcripts may be targeted by a single sRNA, such as *Botrytis cinerea* siR37, which was shown to target *Arabidopsis thaliana FEI2*, WRKY transcription factors and defensins, increasing host susceptibility to infection (Wang *et al*., 2017). Most commonly, ckRNAi‐acting pathogen‐derived sRNAs co‐purify with host AGO1 (Weiberg *et al*., 2013; Dunker *et al*., 2020), with a 5′ uridine that utilises the AGO1 loading bias to drive post‐transcriptional gene silencing against host transcripts (Mi *et al*., 2008); indeed, the importance of host AGO1 function in ckRNAi can be seen in the increased resistance to *B. cinerea* exhibited by Arabidopsis *ago1* mutants (Weiberg *et al*., 2013). Recent reports suggest, however, that some pathogens may manipulate other plant AGOs in ckRNAi – the 23 nt miRNA‐like *Fol*‐milR1 released by *F. oxysporum* f.sp. *lycopersici*, for example, preferentially binds *Solanum lycopersicum* AGO4a to downregulate expression of a host calcium‐binding protein kinase involved in defence signalling (Ji *et al*., 2021). AGO4 – with a 5′ adenosine bias (Mi *et al*., 2008) – predominantly functions in RNA‐directed DNA‐methylation, facilitating transcriptional gene silencing. Whilst cleavage of the target transcript was observed, suggesting post‐transcriptional gene silencing, it was not ruled out that *Fol*‐milR1 also promoted RNA‐directed DNA‐methylation through its association with *Sl*AGO4a, and it may thus be possible that *Fol*‐milR1 facilitates silencing of the host target through two pathways.

**Table 1:** Studies conducted on ckRNAi.

Host species	Direction of sRNA transport	Parasite/mutualist species	Type of interaction	Target gene(s)* or gene ontology (GO) terms	sRNA	Experimentally validated?**	Reference
*Glycine max*	⇐	*Bradyrhizobium japonicum*	Mutualistic	*ROOT HAIR DEFECTIVE 3* (*RHD3a/RHD3b*) *HAIRY MERISTEM 4* (*HAM4a/HAM4b*) *LEUCINE‐RICH REPEAT EXTENSION‐LIKE 5* (*LRX5*)	21 nt tRNA fragments (tRFs) Bj‐tRF001 Bj‐tRF002 Bj‐tRF003	Yes	Ren *et al*. (2019)
*Phaseolus vulgaris*	⇐	*Rhizobium etli*	Mutualistic	*RGA1* (GRAS family TF) *AP2* (*APETALA‐2* –like TF)	21 nt tRNA fragments (tRFs)	No	
*Medicago truncatula*	⇐	*Rhizophagus irregularis*	Mutualistic	*DREPP* (PM protein) *NPC4* (non‐specific phospholipase) *VAPYRIN* (AM symbiotic host‐specific ankyrin repeat protein) NB‐LRR		No	Silvestri *et al*. (2019)
*Medicago truncatula*	⇐	*Gigaspora margarita*	Mutualistic	GO terms: ‘signal transducer activity’, ‘molecular transducer activity’, ‘lipid binding’ AES62408 (chitinase) AES77475 (expansin) AET01158 (putative CCR4‐NOT transcription complex protein)	21 nt sRNAs (including of mycoviral origin)	No	Silvestri *et al*. (2020)
		*Rhizophagus irregularis*		AES62408 (chitinase) AES77475 (expansin) AET01158 (putative CCR4‐NOT transcription complex protein)			
*Eucalyptus grandis*	⇐	*Pisolithus microcarpus*	Mutualistic	*Eucgr.E03170* (CC‐NLR)	miRNA Pmic_miR‐8	No	Wong‐Bajracharya *et al*. (2022)
*Phaseolus vulgaris*	⇐	*Rhizobium tropici*	Mutualistic	*Early Responsive to Dehydration* (*ERD*) *Early NODulin‐Like protein 17* (*ENOD17*) *HEMA1* (glutamyl‐tRNA reductase)	21 nt tRNA fragments (tRFs) Glu‐TTC Met‐CAT2 Met‐CAT3 Trp‐CCA	No	Sánchez‐Correa *et al*. (2022)
*Populus trichocarpa*	⇒	*Laccaria bicolor*	Mutualistic	Transport protein particle complex subunit (TRAPP) 20 K subunits, kinesin light chain	6 19–22 nt miRNAs	No	Mewalal *et al*. (2019)
*Populus trichocarpa*	⇒	*Rhizophagus irregularis*		HMG‐box transcription factor	5 20–24 nt miRNAs		
*Populus deltoides*	⇒	*Laccaria bicolor*		Transport protein particle complex subunit (TRAPP) 20 K subunits, predicted transcription factors, predicted membrane efflux pumps, zinc‐finger proteins	14 20–24 nt miRNAs		
*Arabidopsis thaliana*	⇐	*Botrytis cinerea*	Pathogenic	*PRXIIF* (peroxiredoxin) *MPK1* *MPK2* Wall‐associated kinase	21 nt sRNAs Bc‐siR3.1 Bc‐siR3.2 Bc‐siR5	Yes	Weiberg *et al*. (2013)
*Solanum lycopersicum*	⇐	*Botrytis cinerea*	Pathogenic	*MAPKKK4* (Mitogen‐activated protein kinase kinase kinase)	21 nt sRNA Bc‐siR5	Yes	Weiberg *et al*. (2013)
*Arabidopsis thaliana*	⇐	*Verticillium dahliae*	Pathogenic		99 *At*AGO1‐loaded sRNAs	No	Wang *et al*. (2016)
*Arabidopsis thaliana*	⇐	*Botrytis cinerea*	Pathogenic	*WRK7* *WRKY57* *FEI2* (LRR‐RK) *PMR6* (pectin lyase) *ATG5* (defensin)	Bc‐siR37	Yes	Wang *et al*. (2017)
*Triticum aestivum*	⇐	*Puccinia striiformis* f. sp. *tritici*	Pathogenic	Pathogenesis‐related 2 (PR2) gene *SM638* (b 1,3 glucanase)	miRNA‐like (milR1)	Yes	Wang *et al*. (2017)
*Arabidopsis thaliana*	⇐	*Blumeria graminis* f. sp. *hordei* and f. sp. *tritici*	Pathogenic	GO terms: ‘fatty‐acyl‐CoA transport’, ‘anion transport’, ‘regulation of seed germination’, ‘cell wall macromolecule metabolic process’, ‘organic substance catabolic process’		No	Kusch *et al*. (2018)
*Hordeum vulgare*	⇐	*Blumeria graminis*	Pathogenic	GO terms: ‘macromolecule‐catabolic processes’			
*Triticum aestivum*	⇐	*Blumeria graminis* f. sp. *tritici*	Pathogenic	GO terms: ‘fatty acid catabolism’, ‘fatty‐acyl‐CoA transport’, ‘ubiquinone biosynthesis’, ‘seed germination’			
*Arabidopsis thaliana*	⇐	*Cuscuta campestris*	Pathogenic	*TIR1* *AFB2*, *AFB3*, *BIK1* *SEOR1* (phloem protein) *HSFB4* (transcriptional repressor)	22 nt miRNAs e.g. miR393	Yes	Shahid *et al*. (2018)
*Arabidopsis thaliana*	⇐	*Sclerotinia sclerotiorum*	Pathogenic	*SNAK2* (SNF1‐related kinase) *SERK2* (somatic embryogenesis receptor‐like kinase 2)	22–23 nt TE‐derived sRNAs	Yes	Derbyshire *et al*. (2019)
*Arabidopsis thaliana*	⇐	*Hyaloperonospora arabidopsis*	Pathogenic	*WNK2* (kinase) *AED3* (protease)	21 nt sRNAs RNA2 RNA90	Yes	Dunker *et al*. (2020)
*Brachypodium distachyon*	⇐	*Magnaporthe oryzae*	Pathogenic	*Transcriptional regulator algH* *Myb‐related protein Zm38* Aquaporins RNA helicases *DCL3b* *RGA3*, *RGA4*, *RPP13‐like protein 3* (resistance genes)	20–22 nt sRNAs	No	Zanini *et al*. (2021)
*Solanum lycopersicum*	⇐	*Fusarium oxysporum* f. sp. *lycopersici*	Pathogenic	*FRG4* (calcineurin B‐like‐interacting protein kinase)	23 nt miRNA‐like *Fol*‐milR1	Yes	Ji *et al*. (2021)
*Triticum aestivum*	⇐	*Puccinia striiformis* f.sp. *tritici*	Pathogenic	19 target genes including: TraesCS2D02G510300.1 (NB‐LRR) TraesCS3A02G302100.1 (glutathione *S*‐transferase) TraesCS7B02G299200.1, TraesCS4D02G316900.1 (bZIP transcription factors)	17 20–21 nt sRNAs	Yes	Mueth & Hulbert (2022)
*Malus x domestica*	⇐	*Valsa mali*	Pathogenic	*RLKT1*, *RLKT2* (receptor‐like protein kinases involved in defence signalling)	miRNA‐like *Vm*‐milR1	Yes	Xu *et al*. (2022)
*Arabdopsis thaliana*	⇐	*Verticillium dahliae*	Pathogenic	*MIR157d* – precursor to miR157d Indirectly targeting *SPL13A*, *SPL13B*	24 nt VdrsR‐1 (rRNA‐derived)	No	Zhang *et al*. (2022)
*Solanum lycopersicum*	⇐	*Botrytis cinerea*	Pathogenic	*ATG2* (*Autophagy‐related 2*) *MPKKK4* (Mitogen‐activated protein kinase kinase kinase) *PPR* (*Pentatricopeptide repeat protein*) *ACIF1* (*Avr9/Cf‐9–INDUCED F‐BOX1*)	21 nt sRNAs Bc‐siR3.1 Bc‐siR3.2 Bc‐siR5	Yes	He *et al*. (2023a)
*Hordeum vulgare*	⇐	*Blumeria hordei*	Pathogenic	GO terms: ‘ADP‐binding’, ‘Nucleotide triphosphatase activity’, ‘Microtubule‐severing ATPase activity’, ‘Transport’, ‘Vacuole’	miRNA‐like (milRNAs)	No	Kusch *et al*. (2023)
*Oryza sativa*	⇐	*Xanthomonas oryzae* pv. *oryzicola*	Pathogenic	*JMT1* (Jasmonate methyltransferase)	Xosr001	Yes	Wu *et al*. (2023)
*Gossypium hirsutum*	⇒	*Verticillium dahliae*	Pathogenic	Ca^2+^−dependent cysteine protease (*Clp‐1*) isotrichodermin C‐15 hydroxylase (*HiC‐15*)	miR166 miR159	Yes	Zhang *et al*. (2016)
*Arabidopsis thaliana*	⇒	*Botrytis cinerea*	Pathogenic	BC1G_10728 – *Vps51* (Vacuolar protein sorting 51) BC1G_10508 – *DCTN1* (dynactin subunit) BC1G_08464*‐*Suppressor of Actin (SAC1)–like phosphoinositide phosphatase	TAS1c‐siR483 TAS2‐siR453	Yes	Cai *et al*. (2018)
*Arabidopsis thaliana*	⇒	*Phytophthora capsici*	Pathogenic	*Phyca_554980* (splicing factor)	PPR‐derived secondary siRNAs e.g. siR1310	No	Hou *et al*. (2019)
*Brachypodium distachyon*	⇒	*Magnaporthe oryzae*	Pathogenic	MGG_05023 (chitin deacetylase 1) MGG_09460 (cell wall protein) MGG_11916*‐CAP20* (virulence gene) MGG_05287‐*CON7* (virulence‐related transcription factor) MGG_09055 – *AvrPiz‐t* (effector protein) MGG_04090‐*Sso7* (SNARE protein) MGG_07201‐*YHM2* (a mitochondrial DNA replication protein) MGG_07667‐*ATG17* (autophagy‐related protein)	20–22 nt sRNAs	No	Zanini *et al*. (2021)
*Triticum aestivum*	⇒	*Puccinia striiformis* f.sp. *tritici*	Pathogenic	9 target transcripts including: KNF02052, KNF02053 (glycosyl hydrolase family 26) KNE96707 (60S ribosomal protein L11)	8 18–24 nt sRNAs	Yes	Mueth & Hulbert (2022)
*Arabidopsis thaliana*	⇒	*Verticillium dahliae*	Pathogenic	Ca^2+^−dependent cysteine protease (*Clp‐1*) isotrichodermin C‐15 hydroxylase (*HiC‐15*)	miR166 miR159	Yes	Zhu *et al*. (2022)
*Hordeum vulgare*	⇒	*Blumeria hordei*	Pathogenic	GO terms: ‘protein phosphorylation’, ‘Protein K63‐linked deubiquitination’, ‘ATP‐binding’, ‘RNA Pol II core complex’		No	Kusch *et al*. (2023)

* Discussed, verified or further explored by paper.

** 5′ RACE assay, Parallel Analysis of RNA Ends (PARE), cleavage reporter assays, RNA integrity assays or qRT‐PCR.

It is important to note, however, that the results from Weiberg *et al*., which pioneered the field of plant–microbe ckRNAi research, have recently been contested. Experiments in the *S. lycopersicum*–*B. cinerea* pathosystem, with the knockout of *B. cinerea DCL1* and *DCL2* and the subsequent reduction in predicted ckRNAi‐acting sRNAs, saw no effect on fungal virulence (Qin *et al*., 2023); this was concluded to show that ckRNAi had no significant role in the host–pathogen interaction. However, as was highlighted by a subsequent rebuttal, this deletion of *BcDCL1/2 –* conducted in an unstable *ku70* background – could not be accurately compared to *BcDCL1/2* mutants as generated in Weiberg *et al*. (He *et al*., 2023a); furthermore, this did not eliminate previously identified sRNAs acting in ckRNAi. This, coupled with unsuitable bioinformatics pipelines and lack of experimental validation for the cleavage of candidate host transcripts by *B. cinerea* sRNAs, makes it likely that the majority of globally predicted sRNA–mRNA pairs do not truly interact and therefore the reduction in these sRNAs would have no effect on the host–pathogen interaction (He *et al*., 2023a). This highlights the level of experimental rigour required to explicitly verify or refute the occurrence of ckRNAi in plant–microbe interactions that must be adhered to in future ckRNAi research (Box 2).

Box 2 **Investigating the role of ckRNAi in AM symbiosis**

Results from *in silico* predictions of *R. irregularis* sRNA targets in *Medicago* suggest the possibility of ckRNAi occurring in this symbiosis (Silvestri *et al*., 2019); however, further experimental validation will be required to both confirm its occurrence and elucidate its importance in regulating the mutualism. Similar approaches to studies of pathogenic ckRNAi systems would be appropriate to answer most open questions in this field, such as the profiling of extracellular (PAS) sRNAs and AGO pull‐down assays, which would indicate the fungal‐to‐plant transfer of *R. irregularis* sRNAs and their interaction with the host RNAi machinery. Further analysis such as 5′ RACE cleavage assays would enable the confirmation of target transcript cleavage, which could be correlated with ectopic expression of *R. irregularis* sRNAs in the host to demonstrate the definitive role of sRNAs in driving transcript cleavage. The colonisation phenotype in the latter study would demonstrate the significance of fungal‐to‐plant ckRNAi in AM symbiosis, which could be further shown through phenotyping the AM symbiosis in host plants overexpressing the targets of the *R. irregularis* sRNAs.

Understanding the role of the potential reciprocal plant‐to‐fungus ckRNAi in AM symbiosis would largely be a similar process. Recent *in silico* predictions of *R. irregularis* genes targeted by symbiotically upregulated *Populus* spp. miRNAs suggest that some gene transcripts may be targeted through ckRNAi, although this did not reveal any symbiotically relevant genes and so ckRNAi in AM symbiosis will require further exploration. As highlighted by recent studies of fungal‐to‐plant ckRNAi, a rigorous bioinformatics pipeline, with sufficient stringency in the selection of RNA profiles analysed – for example, those significantly upregulated during the symbiosis in a root‐specific manner, and not total RNA profiles – will be required in this exploration to identify sRNA–mRNA pairs which are more likely to be truly interacting in ckRNAi (He *et al*., 2023a; Qin *et al*., 2023). These recent examples also demonstrate the necessity for experimental validation of sRNA–mRNA interactions, following preliminary *in silico* results, to differentiate between predicted and actual transcript targeting, as has been enabled by RACE assays in many ckRNAi studies (Table 1). This could be further bolstered by analysis of sRNA association with AGOs in the receptive organism, demonstrating the interaction with RNAi machinery required for ckRNAi to occur (He *et al*., 2023a). However, determining the significance of this ckRNAi will be additionally hindered by the current inability to transform *R. irregularis*; this is likely to be overcome using HIGS, which will enable the overexpression or silencing of candidate fungal target genes to determine the effect of modulating candidate gene expression on the symbiosis. These fundamental steps will be required to prove the presence and significance of ckRNAi in AM symbiosis; further experiments, modulating the type and abundance of sRNAs transferred between partners, could explore the potential for manipulating this communication mechanism to enhance symbiotic function.

The possibility has also recently emerged that sRNAs are not the only class of RNA transported in pathosystems, with evidence suggesting that *Ustilago maydis*, a fungal pathogen of maize, transports EV‐associated mRNAs into its host. These mRNAs were enriched in transcripts encoding enzymes involved in nitrogen and glycerolipid metabolism, as well as aromatic amino acid biosynthesis (Kwon *et al*., 2021); this is in line with evidence of host metabolism reprogramming by *U. maydis* (Doehlemann *et al*., 2008), suggesting that mRNA transfer may enable a transfer of the metabolic load of protein synthesis to the host, with the further benefit of immediate delivery of these enzymes into the host. The efficacy of this mechanism has clear advantages in the invasion of host tissue, so although not thus far observed in other plant–microbe interactions, this may change with further study. Alternatively, it may be that this mechanism is unique to *U. maydis* due to its lack of RNAi machinery that prevents the use of ckRNAi to manipulate gene expression in the host (Laurie *et al*., 2008).

### Plant‐to‐pathogen communication

Research into the phenomenon of plant‐to‐pathogen sRNA transport and ckRNAi is comparatively recent, yet examples have been documented for both fungal and oomycete pathogens (Table 1; Fig. 1a). As with the reciprocal ckRNAi event, plant‐driven ckRNAi affects the expression of genes critical to the success of the infection cycle; these may include virulence‐specific components of the secretion system, as in the *Arabidopsis*–*B. cinerea* pathosystem (Cai *et al*., 2018), or splicing factors required for successful reproduction, as in the *Arabidopsis*–*Phytophthora capsici* pathosystem (Hou *et al*., 2019). Intriguingly, recent results from host‐induced gene silencing (HIGS) studies suggest that this means of gene expression regulation by the host may also function in bacterial pathogens, such as *Pseudomonas syringae*, which lack conventional RNAi machinery (Singla‐Rastogi *et al*., 2019); this may be facilitated by host AGO1 proteins taken up alongside the sRNAs, as these have recently shown to bind plant extracellular sRNAs used in ckRNAi (He *et al*., 2021; Box 1). This unexpected result suggests the possibility that plants may be able to use native sRNAs in ckRNAi against a range of pathogens, a new dimension to the plant immune system that merits further investigation.

## III. The roles of sRNAs in mutualistic symbiosis

### Mutualist microbe‐to‐plant communication

As an emerging area of the field, the abundance of *in vivo* studies of the role of ckRNAi in mutualistic interactions is limited; however, there is evidence of ckRNAi between *Medicago truncatula* and the bacterial symbiont *Bradyrhizobium japonicum*, with the production of tRNA fragments (tRFs) capable of driving *Glycine max* transcript cleavage strongly upregulated in *B. japonicum* during root nodulating symbiosis. These tRFs were shown to target root development genes critical in nodule formation such as *ROOT HAIR DEFECTIVE 3* and *HAIRY MERISTEM 4* (Table 1; Fig. 1b); the introduction of target mimics of *Bj*tRFs significantly reduced nodule formation and bacterial colonisation, indicating that the use of ckRNAi to modulate host development is critical to the success of the root nodulating symbiosis (Ren *et al*., 2019). Furthermore, rhizobial tRFs were found to bind *Gm*AGO1, and have been shown to bind host AGO5 in the *Phaseolus vulgaris–Rhizobium tropici* symbiosis, suggesting that host RNAi is manipulated in a similar manner to ckRNAi in pathosystems (Ren *et al*., 2019; Sánchez‐Correa *et al*., 2022).

Thus far, evidence in support of ckRNAi in mycorrhizal symbioses includes the miRNA‐directed cleavage of host nucleotide‐binding leucine‐rich repeat (NB‐LRR) defence gene transcripts in the ectomycorrhizal symbiosis between *Eucalyptus grandis* and *Pisolithus microcarpus* (Fig. 1b). Inhibition of the *Pmic*_miR‐8 miRNA significantly reduced the maintenance of the mycorrhizal association, indicating that such mechanisms carry significant weight in the success of mycorrhizal symbioses (Wong‐Bajracharya *et al*., 2022). The case for ckRNAi in AM symbiosis is supported by *in silico* predictions of sRNA–host transcript pairs which remain to be experimentally validated, in combination with evidence of complete RNAi machinery complements conserved across AM fungi (Lee *et al*., 2018; Silvestri *et al*., 2020) and significantly expanded in species such as *Rhizophagus irregularis* (Dallaire *et al*., 2021). As in pathogenic and ectomycorrhizal systems, the fungal symbiont appears to target host defence genes, but also membrane‐remodelling phospholipases that may permit the formation of the PAM (Silvestri *et al*., 2019, 2020), akin to the development‐regulating role of tRFs in root nodulating symbioses (Ren *et al*., 2019).

### Plant‐to‐microbial mutualist communication

Evidence for reciprocal communication in these symbioses is considerably scarcer than in pathosystems (Table 1), although *in silico* experiments have suggested that *Populus* spp. miRNAs are capable of targeting gene expression in both ectomycorrhizal (*Laccaria bicolor*) and AM (*R. irregularis*) fungal partners (Mewalal *et al*., 2019). These remain to be experimentally verified, and, moreover, do not appear to target symbiotically relevant genes (Table 1); however, given the evidence of many other bidirectional signalling processes involved in establishing and maintaining these symbioses (reviewed in Lanfranco *et al*., 2018), it might be expected that the plant host in fact regulates key functions of its endosymbiont during symbiosis. It is therefore likely that further *in silico* exploration beyond this initial study and, crucially, experimental validation, will reveal true ckRNAi in these symbioses. Whilst it might be argued that the capacity for ckRNAi is limited in the root nodulating symbiosis due to the lack of bacterial RNAi machinery, the evidence of successful HIGS in the *Arabidopsis–P. syringae* pathosystem suggest that plant‐derived sRNAs are capable of driving bacterial gene silencing through an as‐yet unidentified mechanism (Singla‐Rastogi *et al*., 2019). As such, there is the potential for reciprocal ckRNAi in both symbioses.

Host‐induced gene silencing experiments in AM symbiosis also lend support to the idea that RNAs may be transported from plant to fungus and modulate fungal gene expression, with HIGS used to experimentally silence *Rhizophagus* spp. and *Gigaspora* sp. transporters, receptors, effectors and secreted proteins (Helber *et al*., 2011; Kikuchi *et al*., 2016; Tsuzuki *et al*., 2016; Xie *et al*., 2016; Voß *et al*., 2018). Recent evidence from transmission electron microscopy (TEM) and tomography of the plant–fungal interface – the PAM and PAS – indicates the presence of EVs of indeterminate origin in the PAS (Ivanov *et al*., 2019; Roth *et al*., 2019). However, TEM also indicated the fusion of multivesicular bodies with the PAM, suggesting that some EVs, at least, are derived from the plant, and furthermore may be analogous to mammalian EVs that are known to courier diverse RNA and protein cargoes to modulate recipient cell function (Roth *et al*., 2019; reviewed in van Niel *et al*., 2018; Holland & Roth, 2023). Studies of pathogenic ckRNAi implicate the roles of these EVs in sRNA transport (Box 1); thus, there is promising evidence for the transport of sRNAs from plant to fungus at the PAM, which, along with *in silico* evidence, suggests the possibility of ckRNAi as a reciprocal regulatory force in AM symbiosis (Mewalal *et al*., 2019; Fig. 1b).

### Perspectives and future directions

At present, definitive *in vivo* evidence for the role of ckRNAi in regulating AM symbiosis is lacking; however, this can arguably be inferred from the substantial body of literature indicating the use of sRNAs in regulating plant interactions with both microbial mutualists and pathogens. Recent results from *in silico* predictions would suggest that AM fungi may likewise be able use ckRNAi to modulate host defence, as well as aspects of arbuscule development through regulating host membrane remodelling (Silvestri *et al*., 2019, 2020). Given the role of the AM symbiosis in supplying inorganic minerals to the plant in exchange for fatty acids, it could be speculated that the fungus might also target host functions that shift the balance of the exchange in its favour – for example, modulating plant fatty acid transport and biosynthesis, or phosphate metabolism to alter the host's demands and the carbon the fungus receives in return, mimicking native RNAi regulation of phosphate‐related genes (Pandey *et al*., 2018). These results remain to be validated *in vivo* and will require further experimental validation (Box 2).

The same is yet to be experimentally demonstrated reciprocally, although the bidirectional nature of the majority of signalling processes involved in establishing and maintaining AM symbiosis would seem to suggest both the possibility and the necessity of such an event, supported by some initial *in silico* predictions (Luginbuehl & Oldroyd, 2017; Lanfranco *et al*., 2018; Mewalal *et al*., 2019). Such plant‐to‐fungus ckRNAi might therefore likewise enable the plant to regulate the metabolism of the AM fungus, modulating functions such as phosphate or lipid uptake and transport, or the timing of arbuscule development, to control the balance of phosphate–lipid exchange. It may also be the case that ckRNAi in AM symbiosis is necessary for the host to modulate the development of the non‐self organism, as TEM tomography showed the formation of membrane tubules in the paramural space that resemble those observed during invasive hyphal growth of *U. maydis* (Ivanov *et al*., 2019; Roth *et al*., 2019), suggesting aggressive invasion of the host tissue that may need to be curbed by a mechanism such as ckRNAi that has been observed to do the same in pathosystems (Cai *et al*., 2018; Hou *et al*., 2019).

Tantalising results from studies of pathogen–plant ckRNAi also open many avenues for future research; for instance, the loading of endosymbiont sRNAs into host AGO4 proteins opens the prospect of these driving transcriptional gene silencing, possibly allowing long‐term regulation of gene expression in the host through methylation of target genes. Such regulation would have considerable benefits in a mutualism, potentially adapting one or both partners to increase the stability of the interaction; indeed, the importance of DNA methylation in symbiosis has been recently demonstrated in ectomycorrhizal symbiosis, with hypomethylation in the *Populus* sp. host associated with decreased association with the *Laccaria bicolor* mycorrhizal fungus (Vigneaud *et al*., 2023). Determining whether this is observed in the AM symbiosis will be an important avenue for research, especially given that this could be modified to increase the stability of symbioses in key crops. Similarly, the recently proposed transfer of mRNAs between host and pathogenic fungi (Kwon *et al*., 2021) might be more widely distributed across plant–microbial interactions, and could form key mechanisms of both pathogenesis and mutualistic colonisation; however, it may also be the case that this is in fact limited to microbes which lack canonical RNAi machinery, complementing the inability to conduct ckRNAi. Again, verifying this will be critical in expanding our understanding of the communication and manipulation between partners occurring in AM symbiosis, and if present, may form the basis of future augmentation of the symbiosis.

## Competing interests

None declared.

## Author contributions

SQ, ZG and RR conceptualised the manuscript; SQ and RR wrote the manuscript.
